# Effects of 8-Week Online, Supervised High-Intensity Interval Training on the Parameters Related to the Anaerobic Threshold, Body Weight, and Body Composition during Pregnancy: A Randomized Controlled Trial

**DOI:** 10.3390/nu14245279

**Published:** 2022-12-11

**Authors:** Hongli Yu, Rita Santos-Rocha, Łukasz Radzimiński, Zbigniew Jastrzębski, Iwona Bonisławska, Andrzej Szwarc, Anna Szumilewicz

**Affiliations:** 1 Department of Sport, Gdansk University of Physical Education and Sport, 80-336 Gdansk, Poland; 2 Sport Sciences School of Rio Maior (ESDRM), Polytechnic Institute of Santarém, 2001-904 Rio Maior, Portugal; 3 Interdisciplinary Centre for the Study of Human Performance (CIPER), Faculty of Human Kinetics, University of Lisbon, 1649-004 Lisbon, Portugal; 4 Department of Health and Natural Sciences, Gdansk University of Physical Education and Sport, 80-336 Gdansk, Poland; 5 Department of Physical Education and Social Sciences, Gdansk University of Physical Education and Sport, 80-336 Gdansk, Poland

**Keywords:** pregnancy, high-intensity interval training, body composition, anaerobic exercise capacity, anaerobic threshold

## Abstract

We aimed to assess the effects of an 8-week, online high-intensity interval training (HIIT) program on the parameters related to the anaerobic threshold (AT), body weight, and body composition in pregnant women. A total of 69 Caucasian women with an uncomplicated singleton pregnancy (age: 31 ± 4 years; gestational age: 22 ± 5 weeks; mean ± standard deviation) were randomly allocated to either an 8-week HIIT program (HIIT group) or to a comparative 8-week educational program (EDU group). Our most important finding was that even with the 8-week progression of pregnancy and physiological weight gain, the HIIT group maintained the same level of parameters related to AT: volume of oxygen at the AT (VO_2_/AT), percentage of maximal oxygen uptake at the AT (%VO_2max_/AT), and heart rate at the AT (HR/AT). In contrast, in the EDU group we observed a substantial deterioration of parameters related to the AT. The HIIT intervention substantially reduced the fat mass percentage (median: 30 to 28%; *p* < 0.01) and improved the total fat-free mass percentage (median: 70% to 72%; *p* < 0.01). In the EDU group, the body composition did not change significantly. An online, supervised HIIT program may be used to prevent the pregnancy-related risk of excessive weight gain and reduction in exercise capacity without yielding adverse obstetric or neonatal outcomes.

## 1. Introduction

The National Center for Health Statistics indicated that maternal mortality has increased by 33% in the second trimester and 41% in the third trimester since the onset of the COVID-19 pandemic [1]. These changes may be attributed to conditions directly related to COVID-19 such as respiratory infections or conditions aggravated by viruses such as hypertension, diabetes, and cardiovascular disease [1].

Obesity in pregnancy is associated with many comorbidities and disorders, including a higher rate of COVID-19 infection and its complications [2]. In addition, obesity significantly reduces the maximal sustained exercise capacity with an earlier reach of the anaerobic threshold (AT) [3]. The AT refers to the moment of metabolism shift during exercise when the oxygen consumption above which aerobic energy production is supplemented by anaerobic mechanisms causes a sustained increase in lactate and metabolic acidosis [4]. This index has been associated with exercise capacity, cardiorespiratory fitness, and surgery risk, making it a useful parameter for the development, implementation, and evaluation of exercise programs [5].

Maternal body composition undergoes profound adaptive changes during pregnancy. Both fat mass and lean body mass grow differently, and excessive weight gain is relatively prevalent. The percentage of fat mass (%FM) was strongly associated with gestational diabetes risk and markers of cardiovascular health in pregnancy [6]. Exercise has beneficial effects on the AT and thereby in patients with obesity, hypertension, diabetes and cardiovascular disease [7,8,9,10,11]. However, many expectant mothers avoid or considerably reduce their usual exercise routine due to the fear of potential risks [12]. According to the current evidence-based guidelines [13,14], there are no known risks associated with moderate-intensity exercise in women with uncomplicated pregnancies. Furthermore, vigorous exercise during pregnancy among women who are well trained prior to conception has no negative effects on the procession of pregnancy, labor, or the unborn child [15]. Aerobic exercise can improve aerobic fitness in pregnant women, promote fat burning, and delay elevations in blood lactate levels during graded exercise testing [7,16,17].

Sports fitness training, particularly high-intensity interval training (HIIT; brief bouts of vigorous exercise interspersed with intervals of rest or active recovery), has recently attracted the attention of researchers [18]. HIIT has been demonstrated to not only improve cardiovascular function but also significantly increase mitochondrial activity in the skeletal muscle, glucose and lipid metabolism, and overall body composition [19].

A minimum of 150 min per week of moderate-to-vigorous exercise during pregnancy is safe and recommended in the absence of obstetric or medical complications or contraindications by credible gynecology, obstetrics, and sports medicine institutes, including the World Health Organization [20,21,22,23]. Yoga and slow walking are common during pregnancy but require a considerable time commitment and training duration to be effective [12]. As a time-efficient alternative, HIIT has evolved into a training approach with the potential to burn fat and enhance the AT in both healthy individuals and patients, including those with cardiovascular disease [24], cancer [25], or obesity [26]. Notably, most studies have involved older adults, women who have already undergone menopause, and pregnant elite athletes. There are limited data on the effectiveness of HIIT in inducing weight loss, body composition, and AT changes in pregnant women who are non-athletes and were inactive before pregnancy [14].

To address the aforementioned concerns, this study aimed to: (1) evaluate the effects of an 8-week HIIT program on selected parameters related to the anaerobic threshold and body composition during pregnancy; and (2) examine the relationship between the characteristics of the exercise intervention and changes in the selected parameters related to the anaerobic threshold and body composition.

## 2. Materials and Methods

### 2.1. Ethics, Recruitment, and Flow of the Participants through the Study

This study was conducted at the Laboratory of Physical Effort and Genetics in Sport at the Gdansk University of Physical Education and Sport in Poland in 2021. All procedures were performed in accordance with the principles outlined in the Declaration of Helsinki of the World Medical Association (WMA) and approved by the Bioethics Commission of the District Medical Chamber in Gdansk (KB-8/21). The entire research protocol was registered with ClinicalTrials.gov (NCT05009433) on 17/08/2021 and was entitled “HIIT vs. MICT During Pregnancy and Health and Birth Outcomes in Mothers and Children (HIIT Mama)”. After the trial began, no marked methodological adjustments were made. The study adhered to the principles of openness, transparency, reproducibility, and the CONSORT standards [27].

This randomized controlled trial was conducted on 69 Caucasian women with uncomplicated singleton pregnancies ([mean ± standard deviation] age: 31 ± 4 years, gestational age: 22 ± 5 weeks) who consented to participate in the study after receiving our mass media invitation.

The eligibility criteria for both groups were as follows: (1) correct course of gestation confirmed during the standard obstetric examination for each pregnant woman in accordance with national law; (2) week of gestation not higher than 28 in order to be able to attend the entire intervention; (3) proficiency in the Polish language; and (4) any age. The exclusion criteria were as follows: (1) contraindications to increased physical exertion or other situations that could adversely affect the health or safety of the participants or the quality of the gathered data; (2) multiple pregnancy; (3) daily alcohol consumption; (4) lack of a tablet or computer with Internet access. Following a thorough explanation of all procedures and the potential risks involved, all participants signed an informed consent form before starting the baseline tests and interventions. All participants continued to receive routine obstetric care throughout the trial. Additionally, we asked them to comply with the recommendations for a healthy diet for pregnant women during the study.

At baseline, there were 35 pregnant women randomly allocated in the high-intensity interval training group (HIIT group). One participant attended only 3 classes and resigned from the intervention due to family commitments. Five participants from the HIIT group were excluded from analysis, because they attended less than 70% of the HIIT sessions planned for the 8-week exercise program (even though they underwent the post-intervention assessments). The reasons for their absence from the HIIT sessions were: busy with study or work (*n* = 2); busy with taking care of an older child (*n* = 2); or had an infection (*n* = 1). Additionally, we excluded from the analysis one participant who did not exercise with the recommended intensity during the HIIT sessions. It must be underlined that none of the HIIT participants were absent due to exercise-related health issues or because they considered the HIIT sessions too intensive for them.

We invited 34 pregnant women to the comparative group, which participated in the 8-week educational program (EDU group). Eleven women did not complete the intervention (i.e., they did not undergo the post-intervention assessments) due to the following reasons: not interested in continuing the program (*n* = 4); preterm birth (*n* = 1); had to take medications that could influence the study outcome (*n* = 1); not feeling well on the day of the second assessment (*n* = 2); or a lack of support from the obstetric care providers to continue the program (*n* = 2). One woman did not provide a reason (*n* = 1).

**Figure 1:**
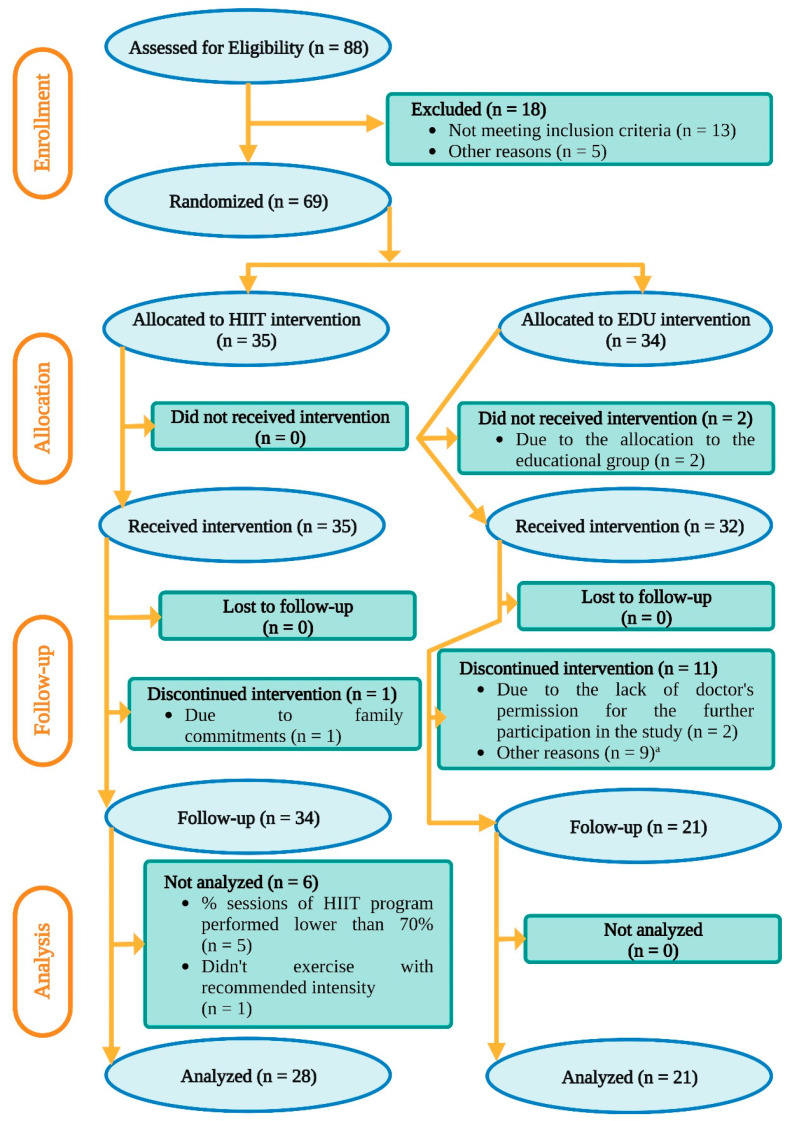
Flowchart of participant selection. EDU: education, HIIT: high-intensity interval training. ^a^ Not interested in continuing the program (*n* = 4); preterm birth (*n* = 1); taking medications that could influence the glucose level or lipid metabolism (*n* = 1); not feeling well on the day of the second assessment (*n* = 2); or did not provide a reason (*n* = 1).

Finally, 49 pregnant women were included into the analysis: 28 from the HIIT group and 21 from the EDU group. The flowchart of participant flow through the study is illustrated in Figure 1.

As part of the characteristics of the study participants at the recruitment stage, we collected their demographic data and measured the level of physical activity using the short form of the International Physical Activity Questionnaire [28]. This questionnaire, which has shown acceptable measurement properties, provides information on weekly physical activity (PA) levels in multiples of the resting metabolic rate (MET). Based on the IPAQ outcomes, we categorized the pregnant participants using three levels (categories) of PA: low (inactive participants), moderate (accumulating a minimum recommended level of PA), and high (exceeding the minimum recommended level of PA) [29,30].

### 2.2. Cardiopulmonary Exercise Test

The cardiopulmonary exercise test (CPET) was conducted according to recommendations by the American Thoracic Society/American College of Chest Physicians using a cycle ergometer with an electronically regulated load (Viasprint 150P; Bitz, Germany) and a pulmonary gas analyzer (Oxycon Pro; Erich Jaeger GmbH, Hoechberg, Germany) [31]. All tests were calibrated according to the manufacturer’s instructions. A data point for every 15 s period was calculated by averaging the breath-by-breath data. The women sat on a chair for 5 min with a silicon face mask for breathing adaptation before the actual test. After the adaptation period, the women began to warm up by cycling for 4 min with a relative load of 0.4 W·kg^−1^ of body mass. When the participants had warmed up, the load was increased by 0.2 W·kg^−1^ per minute until they refused. In preparation for the test, the women were encouraged to cycle up to the limit of their physical capacity. They were also informed that they could stop the test at any time. The participants rested for 3 min after they finished cycling. We used the same CPET protocol before and after the 8-week exercise program. At these two timepoints the number of applied Watts was related to individual participant’s body weight (after 8 weeks the number of Watts was adjusted to the increased body weight).

The maximal oxygen uptake (VO_2max_) was defined as the volume of oxygen consumed at maximal exertion sustained for 15 s. The AT was determined by utilizing a modified V-slope method and the ventilatory equivalent (VE) method [32]. The aerobic threshold (AerT) was determined by plotting the VE as a function of oxygen consumption (VE/VO_2_). The volume of oxygen (VO_2_) at which the lowest VE/VO_2_ values were observed was defined as the AerT [33,34,35]. We set an individual heart rate at the aerobic threshold (HR/AerT) for each participant.

To determine the changes in the parameters related to the anaerobic thresholds of the participants, we analyzed the following parameters: volume of oxygen at the anaerobic threshold (VO_2_/AT), heart rate at the anaerobic threshold (HR/AT), percentage of maximal oxygen uptake at the anaerobic threshold (%VO_2max_/AT), time between the HR/AerT and HR/AT (time during the CPET from the moment when the aerobic capacity was fully used to the threshold when anaerobic exercise started to dominate), and time above the HR/AT (time from the moment when anaerobic exercise started to dominate up to exhaustion and termination of the test).

It should be pointed that cycle ergometer exercise tests are usually associated with significantly lower values of cardiopulmonary parameters (including HR_max_ and HR/AT) compared to treadmill exercise tests [36]. However, due to the higher risk of falling and higher load on the pelvic floor muscles during the maximal test performed by walking or jogging on the treadmill, we decided to use the cycle ergometer test. The data on selected parameters related to AT were collected in the same conditions before and after the 8-week interventions. Therefore, the analysis of changes in these parameters was trustworthy. We are aware that due to the above-mentioned characteristics of the exercise tests, the HR values given to the participants as HR/AT could be underestimated. Nevertheless, these values were set using the same methodology, which provided the same level of underestimation (if any) for all participants.

### 2.3. Body Composition

We measured the participants’ body mass and composition via bioelectrical impedance analysis using InBody 720 (InBody USA, Cerritos, CA, USA). The body mass index (BMI) was calculated as follows: weight in kilograms/height in meters squared. The reliability of InBody 720 was previously proven by McLester et al. [37]. Before and after the HIIT and EDU interventions, we utilized the standard InBody 720 to record and analyze the total fat mass percentage (%FM) and total fat-free mass percentage (%FFM).

### 2.4. High-Intensity Interval Training

Our preparation for the HIIT intervention used in this trial was preceded by a thorough analysis of the current guidelines for exercise during pregnancy published by credible obstetrics, gynecology, or sports medicine institutions [13]; an analysis of recommendations on prenatal exercise program design and implementation [13,38]; and a review of available data on HIIT during pregnancy [14].

**Figure 2:**
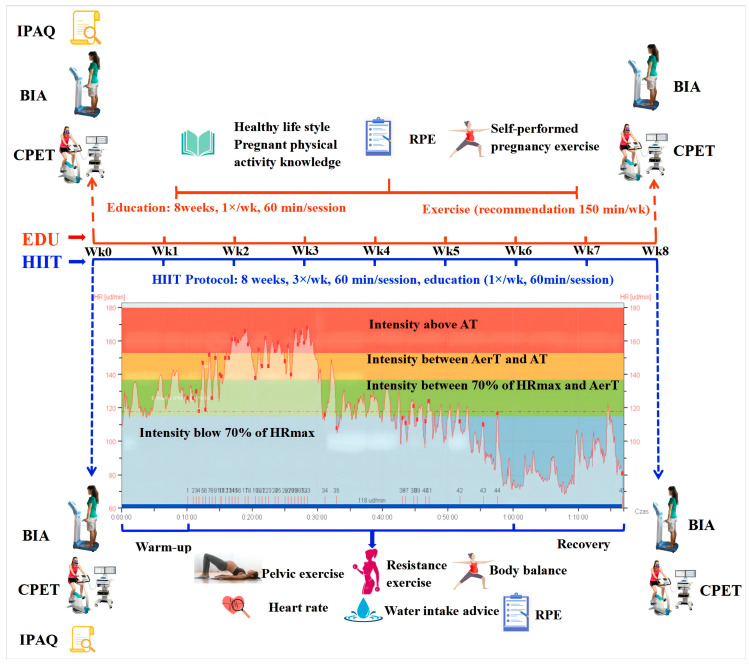
Schematic representation of the study protocol. AerT: aerobic threshold, AT: anaerobic threshold, BIA: bioelectrical impedance analysis, CPET: cardiopulmonary exercise test, EDU: education, HIIT: high-intensity interval training, HR_max_: maximal heart rate, IPAQ: International Physical Activity Questionnaire, RPE: rating of perceived exertion.

The HIIT intervention consisted of three training sessions per week for 8 weeks with each session lasting 60 min. Seven to ten minutes were spent on a warm-up and instruction on how to perform the main exercises. The main part of the session consisted of a high-intensity interval exercise, which lasted 15–20 min (Figure 2). The HR/AT was calculated for each participant using a progressive maximal exercise test and set at 87 ± 5% of the maximal heart rate on average. The participants were instructed to use a heart rate monitor (Polar RS400, Kempele, Finland) and train at intensities that exceeded the HR/AT for as long as they felt comfortable during the workout interval. We assumed that some of them would not be able to exercise above the AT in all workout intervals, inter alia due to the fear of endangering the baby or feelings of tiredness. However, even though they spent more time below the AT (only trying to reach this level) during the sessions, we can define our intervention as HIIT. According to the definition by Wood et al., HIIT protocols should be based on short work intervals (<60 s–8 min) of vigorous (70–90% maximal heart rate or 14–16 of the 6–20 Borg’s rate of perceived exertion—RPE) to high intensity (≥90% maximal heart rate or ≥17 of the 6–20 RPE) interspersed with active (40–70% maximal heart rate or 8–13 of the 6–20 RPE) or passive (cessation of movement) recovery periods (of 1–5 min) [39]. The individual HR values from all sessions were recorded and analyzed after the completion of the intervention.

Additionally, the 0–10 Borg Rating of Perceived Exertion (RPE) Scale [40] and the Talk Test [41] were used to assess the exercise intensity. In order to monitor the participants’ well-being and their acute response to exercise, we asked them to complete individual Exercise Monitoring Cards after each exercise session. The Exercise Monitoring Cards contained the following information: date of exercise session, form of physical activity (participants were asked to enter all forms of physical activity also individually taken; e.g., walking, cycling), the duration of exercise, subjective assessment of exercise intensity at 0–10 RPE scale, rest time after exercise, well-being during or after exercise on day of classes, any comments, and the reason for absence (applicable to HIIT sessions) [13].

The workout intervals included exercises that targeted the major muscle groups (e.g., lunges, squats, jumps, or combinations with upper body movements). The ratio of exercise to rest was set at 1:2, 1:1, or 2:1 depending on the ability of the participant, stage of pregnancy, and progression of training. The duration of each exercise ranged from 30 to 60 s with an equal-length rest break interval. We present the proportions of workout and rest intervals in the Table 1. After the interval portion of training, the participants performed 5–10 min of resistance, neuromotor (i.e., body balance), postural, and stretching exercises. The cool-down consisted of birth preparation and pelvic floor muscle exercises (i.e., birth position and breathing exercises; 5–10 min) along with relaxation and visualization of pregnancy and labor (5–15 min) (Figure 2). There was no workout equipment and the only resistance came from the participants’ body weight. This exercise program was tailored to the requirements and capacities of the pregnant women based on diagnostic exercise test results. It was offered to the pregnant women regardless of their fitness level, athletic ability, or motor skills [13].

The HIIT sessions were held online through MS Teams from 9:30 a.m. to 10:30 a.m. on Mondays, Wednesdays, and Fridays except for one Monday that was a holiday (23 sessions in total). Even though we communicated with the participants through the Internet, all session were supervised in real time. We monitored the participants’ well-being and exercise performance, corrected their technical mistakes, and asked for feedback. Therefore, for our intervention we used the term “online, supervised exercise program”. On average, the participants attended 18 ± 5 sessions, which accounted for 78% of the entire training program. The HIIT group was allowed to perform additional exercise sessions as desired. The average number of additional sessions was 12 (median) with a range (min–max) of 0 to 39, while the average intensity was 5 (median) with a range (min–max) of 0 to 7 on the Borg RPE Scale. Before the program, the women were guided through the use of MS Teams to attend the online sessions and the safety precautions for exercising at home, which included the safe arrangement of space at home and communication guidelines in the case of an accident or worsening in health. The HIIT intervention was combined with an educational lesson once a week. The sessions were conducted by the primary researcher, who is a qualified as a Pregnancy and Postnatal Exercise Specialist according to the European educational standards [42] and is additionally educated in terms of online coaching in accordance with the European Lifelong Learning Qualification “Online Provision of Fitness Services” [43]. We utilized email and phone communication to maintain program adherence.

The educational intervention focused on a healthy lifestyle, physical exercise throughout the perinatal period, and specific issues in pregnancy and parenthood. It followed the same structure as the HIIT intervention (Figure 2). Online, synchronous educational classes were held once each week for a total of 8 weeks. We encouraged the EDU group to engage in physical activity on their own and attain at least the minimum level of physical activity recommended (a minimum of 150 min per week of moderate-to-vigorous intensity physical activity) (Figure 2). The group was asked to record a log of all their physical activity including daily activities (i.e., cleaning the house, shopping, or gardening) that lasted at least 10 min and any structured exercise sessions. The Talk Test and Borg RPE Scale were employed to measure exercise intensity instead of heart rate monitoring. We suggested a level of exercise intensity at which they had a noticeable increase in breathing frequency. The women reported an average of 20 (median) bouts of physical activity with a mean intensity of 5 (median) on the Borg RPE Scale. The EDU group was also asked to complete the individual Exercise Monitoring Cards after each session.

Two months after delivery, we asked all participants about their childbirth outcomes using the same online questionnaire as in our previous study [44]. We collected data that included, among others, the gestational age at birth, type of delivery (nonoperational vaginal delivery, operational gestational delivery, or cesarean section), labor induction, labor augmentation, perineal lacerations, anesthetics used, and newborn’s weight at birth and APGAR scores.

### 2.5. Statistical Analysis

The total sample size was predetermined via a priori and sensitivity analyses using G*×Power version 3.1.3. We expressed most data as means ± SDs and tested the normality of the data using the Shapiro–Wilk test. We presented non-normally distributed data as medians and ranges. The chi-square test was used to compare the non-parametric demographic characteristics between the EDU and HIIT groups. We used one-way ANOVA to compare the changes between the groups after interventions, and the value of Cohen’s *f* was used to represent effect size (effect size conventions: small, *f* = 0.1*;* medium, *f =* 0.25; large, *f* = 0.4). Paired Wilcoxon tests were used to evaluate the changes before and after the HIIT intervention if data were not normally distributed (i.e., VO_2_/AT and time above the HR/AT), and the value of Cohen’s *d* was used to represent effect size (effect size conventions: small, *d* = 0.2; medium, *d* = 0.5; large, *d* = 0.8). We used Spearman’s correlation coefficients to analyze the association of the changes in parameters related to AT and the changes in the body composition with the characteristics of the interventions. The significance level was set at *p* ≤ 0.05. We performed all statistical analyses using OriginPro 2021 (version 9.8.0.200, OriginLab).

## 3. Results

**Figure 3:**
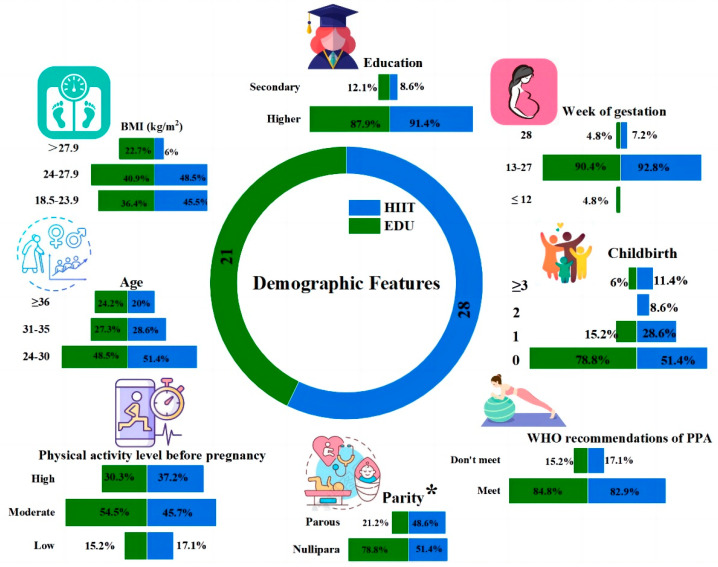
Comparison of the baseline demographic characteristics between the HIIT (*n* = 28) and EDU (*n* = 21) groups, BMI: body mass index, EDU: education, HIIT: high-intensity interval training. The analysis was conducted using the chi-square test (* *p* < 0.05).

A total of 49 participants were included for analysis (*n* = 28 in the HIIT group, *n* = 21 in the EDU group). Apart from parity (*p* < 0.05, Cohen’s *f* = 0.36), none of the baseline demographic variables significantly differed between the groups (Figure 3). The participants in the HIIT and EDU group were in their 20 ± 4 and 23 ± 5 weeks of gestation, respectively. The difference was not statistically significant (*p* = 0.12). It also did not have clinical significance. The development of pregnancy proceeds at an individualized pace and its physiological termination is considered to be the time between the 38th and 42nd week of gestation. Therefore, the group of pregnant women with a difference of 3 gestational weeks can be considered homogeneous in this respect.

### 3.1. Parameters Related to the Anaerobic Threshold

**Figure 4:**
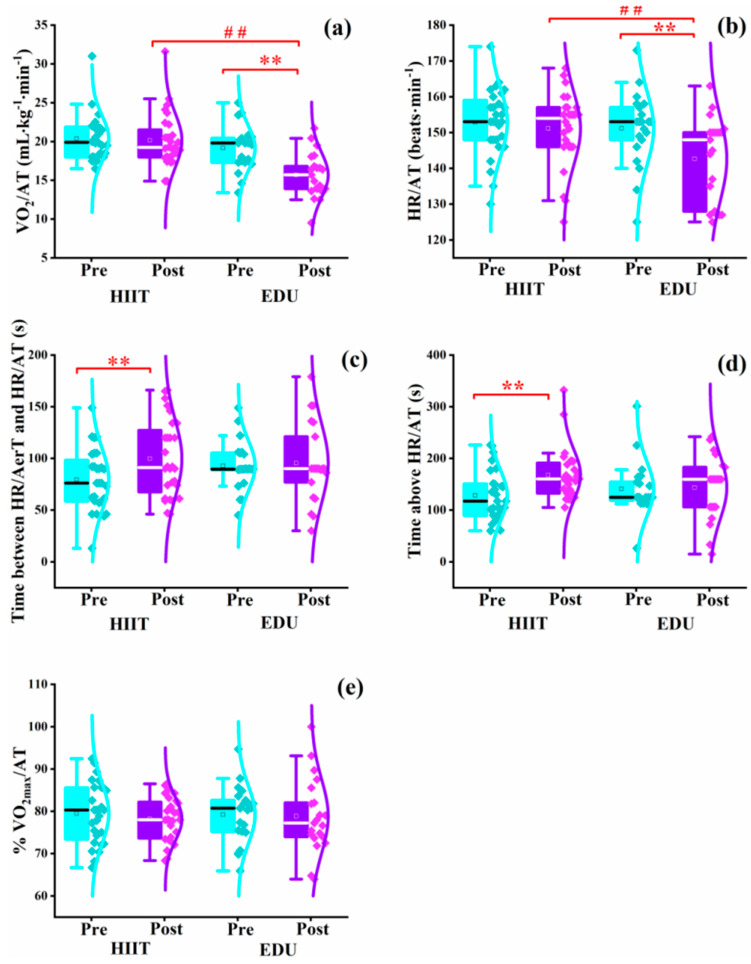
Box charts (**a**–**e**) showing the entire distribution of raw data (rhombus) and the median value (central line) of the parameters related to the anaerobic threshold before and after the 8-week HIIT (*n* = 28) and educational interventions (*n* = 21). EDU: education, HIIT: high-intensity interval training, HR/AerT: heart rate at the aerobic threshold, HR/AT: heart rate at the anaerobic threshold, VO_2_/AT: maximal oxygen uptake at anaerobic threshold, %VO_2max_/AT: percentage of maximal oxygen uptake at the anaerobic threshold. Data were analyzed using one-way ANOVA (^##^ *p* < 0.01) and a paired Wilcoxon test (** *p* < 0.01).

The baseline parameters related to the AT did not significantly differ between the HIIT and EDU groups (Table 2). After 8 weeks, the values of VO_2_/AT were significantly higher in the HIIT group than in the EDU group (HIIT group: 20.15 ± 3.47 mL⸳kg^−1^⸳min^−1^ vs. EDU group: 15.67 ± 2.89 mL⸳kg^−1^⸳min^−1^, *p* < 0.01, Cohen’s *f* = 0.73; Figure 4a). Similarly, the values of HR/AT were significantly higher in the HIIT group than in the EDU group after the interventions (HIIT group: 151 ± 10 beats⸳min^−1^ vs. EDU group: 143 ± 12 beats⸳min^−1^ vs., *p* < 0.01, Cohen’s *f* = 0.48; Figure 4b).

We observed a further beneficial outcome of the HIIT intervention: the time between the HR/AerT and AT (pre- to post-intervention median: 76 to 92 s, z = 2.76, *p* < 0.01, Cohen’s *d* = 0.43; Figure 4c) and the time above the HR/AT (pre- to post-intervention median: 117 to 160 s, z = 3.59, *p* < 0.01, Cohen’s *d = 0.83*; Figure 4d) were considerably improved in this group after 8 weeks. In contrast, in the EDU group we noticed worse values of some parameters related to the AT. The values of VO_2_/AT and HR/AT significantly decreased in the EDU group (pre- to post-intervention median: 19.7 to 15.3 mL⸳kg^−1^⸳min^−1^, z = 3.92, *p* < 0.01, Cohen’s *d* = 1.94 (Figure 4a); 150.5 to 140.5 beats⸳min^−1^, z = 3.41, *p* < 0.01, Cohen’s *d* = 1.39 (Figure 4b)).

### 3.2. Body Composition

**Figure 5:**
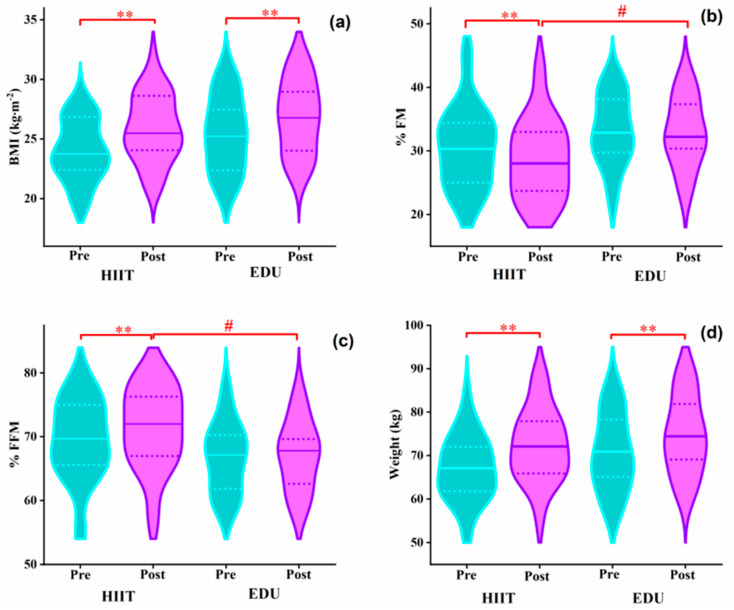
Violin plots (**a**–**d**) showing the entire distribution of the median (central line) and interquartile range (lower and upper lines) of the body composition before and after 8 weeks of the HIIT and educational interventions. BMI: body mass index, EDU: education, HIIT: high-intensity interval training, %FFM: fat-free mass percentage, %FM: fat mass percentage. Data were analyzed using one-way ANOVA (^#^ *p* < 0.01) and a paired Wilcoxon test (** *p* < 0.01).

The baseline body composition did not significantly differ between the HIIT and EDU groups (Table 3). Interestingly, in the HIIT group the %FFM substantially increased after the 8-week intervention (from a median of 70% to 72%; z = 4.25, *p* < 0.01, Cohen’s *d* = 1.05; Figure 5c) and the %FM substantially decreased after 8 weeks of HIIT program (from a median of 30% to 28%; z = 4.25, *p* < 0.01, Cohen’s *d =* 1.05; Figure 5b).

What is more, after the interventions, in the HIIT group the %FM was significantly lower (respectively: 28% and 32%; *p* < 0.05, Cohen’s *f* = 0.34; Figure 5b) and the post-intervention %FFM was higher than in the EDU group (respectively: 72% vs. 68%; *p* < 0.05, Cohen’s *f* = 0.34; Figure 5c).

As expected due to the progression of the pregnancies, the BMI and body weight were significantly increased in both groups after the 8-week interventions The outcomes for BMI were: in the HIIT group, from a median of 23.74 to a median of 25.48 kg·m^−2^, z = 4.70, *p* < 0.01, Cohen’s *d* = 3.73; in the EDU group, from a median of 25.23 to 26.77 kg·m^−2^, z = 3.98, *p* < 0.01, Cohen’s *d* = 2.05 (Figure 5a). The outcomes for body weight were: in the HIIT group, from a median of 67.1 to 72.1 kg, z = 4.70, *p* < 0.01, Cohen’s *d* = 3.95; and in the EDU group, from a median of 70.9 to 74.4 kg, z = 3.98, *p* < 0.01, Cohen’s *d* = 2.06; (Figure 5d).

### 3.3. Relationship of the Body Composition and the Parameters Related to the Anaerobic Threshold with the Intervention Characteristics

**Figure 6:**
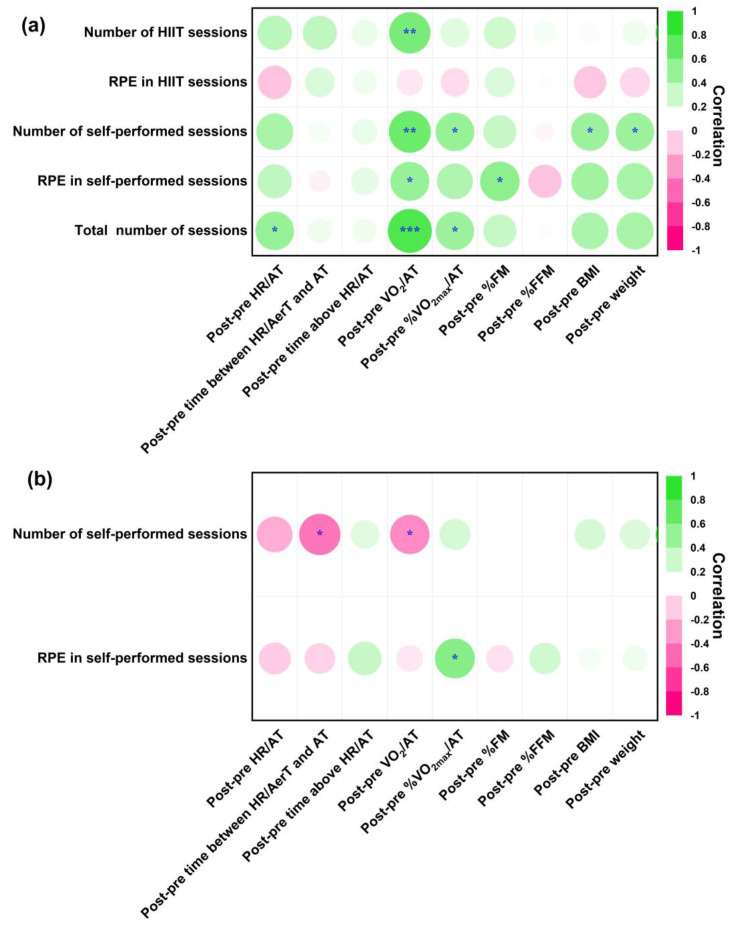
Heat maps of Spearman’s correlation coefficients of the changes in the body composition and parameters related to the anaerobic threshold and intervention characteristics in the HIIT (**a**) and EDU groups (**b**). AerT: aerobic threshold, BMI: body mass index, EDU: educational, HIIT: high-intensity interval training, HR/AerT: heart rate at the aerobic threshold, HR/AT: heart rate at the anaerobic threshold, RPE: rating of perceived exertion, VO_2_/AT: maximal oxygen uptake at anaerobic threshold, %FFM: fat-free mass percentage, %FM: fat mass percentage, %VO_2max_/AT: percentage of maximal oxygen uptake at the anaerobic threshold. *** *p* < 0.001; ** *p* < 0.01; * *p* < 0.05. A darker color indicates a stronger correlation and vice versa; green shows a positive association, whereas red shows a negative association.

The Spearman’s correlation coefficients between the changes in the parameters related to the anaerobic threshold (%VO_2max_/AT, HR/AT, time above the HR/AT, VO_2_/AT, and time between the HR/AerT and HR/AT) and body composition (BMI, %FM, and %FFM) and the characteristics of the intervention (number of HIIT sessions, number of self-performed sessions, and RPE) in the HIIT and EDU groups are shown in Figure 6.

In the HIIT group, the number of HIIT sessions was positively correlated with the change in the VO_2_/AT (r = 0.53, *p* < 0.01), and the number of self-performed sessions was positively associated with the changes in the VO_2_/AT (r = 0.58, *p* < 0.01), %VO_2max_/AT (r = 0.40, *p* < 0.05), BMI (r = 0.38, *p* < 0.05), and weight (r = 0.40, *p* < 0.05). Interestingly, the higher the intensity of self-performed sessions as measured by RPE scale, the higher the increase in the values of VO_2_/AT (r = 0.42, *p* < 0.05) and %FM (r = 0.46, *p* < 0.05). What is more, the more exercise sessions women performed in total, the more significant the changes in the VO_2_/AT (r = 0.68, *p* < 0.001), HR/AT (r = 0.40, *p* < 0.05), and %VO_2max_/AT (r = 0.40, *p* < 0.05) were.

In the EDU group, the number of self-performed sessions was negatively correlated with the changes in the time between the HR/AerT and HR/AT (r = −0.53, *p* < 0.05) and VO_2_/AT (r = −0.45, *p* < 0.05), while the RPE in the self-performed sessions was positively correlated with the %VO_2max_/AT (r = 0.47, *p* < 0.05).

Neither in the HIIT nor in the EDU group did we observe any adverse effects of our interventions on the development of pregnancy, childbirth, or neonatal outcomes The premature birth that was noted by one of the participants from the EDU group was not related to physical activity or any other lifestyle factors.

## 4. Discussion

In this study, we examined the effects of an 8-week online, supervised HIIT intervention on parameters related to the anaerobic threshold (AT) and body composition in pregnant women. One of our most important findings was that in the HIIT group, the parameters related to the AT were better or maintained at the same level after the 8-week intervention even with the progression of pregnancy and weight gain. Interestingly, the HIIT intervention reduced the %FM and improved the lean body mass of the pregnant women. In contrast, in the EDU group we observed a substantial deterioration in parameters related to the AT, and the body composition did not change significantly. Consequently, our online HIIT program may be used to prevent the pregnancy-related risk of excessive weight gain and reduction in exercise capacity as well as during the COVID-19 pandemic and in the case of limited access to sport facilities.

### 4.1. Parameters Related to the Anaerobic Threshold

Sloth et al. [45] found that short-term low-volume HIIT (2–8 weeks) was efficacious in increasing the VO_2_/AT in healthy but sedentary adults as well as recreationally active adults and that the 4-week HIIT increased the VO_2_/AT by 13% in healthy older adults [46]. In addition to the VO_2_/AT, we examined other parameters related to the AT that included the HR/AT, VO_2_/AT, %VO_2max_/AT, time between the HR/AerT and HR/AT, and time above the HR/AT. We observed that the VO_2_/AT, HR/AT, and %VO_2max_/AT of the healthy pregnant women did not increase over the 8 weeks of HIIT (Figure 4). Nevertheless, these outcomes seemed to be beneficial when considering the progression of pregnancy and physiological weight gain during these 8 weeks of intervention. Thus, the effectiveness of HIIT in pregnant women should not be ignored. Soma-Pillay et al. [47] reported that the cardiorespiratory workload increased with fetal growth and oxygen consumption during pregnancy. In a study by Melzer et al. [48], cardiorespiratory fitness and the AT decreased during pregnancy as the body weight and cardiorespiratory load increased. In our study, the values of HR/AT and VO_2_/AT significantly decreased in the EDU group but did not deteriorate in the HIIT group (Figure 4). What is more, after the interventions, the values of HR/AT and VO_2_/AT were significantly higher in the HIIT group than in the EDU group.

Our observations may encourage pregnant women, including non-athletes, to participate in high-intensity interval training. This in turn may broadly support women’s health and the normal course of their pregnancies because increasing the AT is associated with improved exercise capacity and cardiovascular fitness and a decreased risk of maternal mortality during the COVID-19 pandemic [3]. Our findings were in line with the studies by other authors who also noted beneficial effects of prenatal HIIT on cardiopulmonary outcomes [14].

### 4.2. Body Weight and Composition

The BMI and weight considerably increased in both HIIT and EDU groups but remained within the normal range specified for pregnancy. Our outcomes were similar to the findings of Ong et al. [49]. The Institute of Medicine recommends that women with a normal pre-pregnancy BMI increase their weight by approximately 5–6 kg during their second and third trimesters [50]. Much of the weight gain during the second trimester is attributed to physiological changes (e.g., increased blood volume, uterus size, breast volume, and fat storage) [51]. Despite regular exercise, pregnancy weight and BMI continue to increase throughout pregnancy [52].

One of the key findings of our study was that the %FM significantly decreased after the HIIT intervention (Figure 5). It is possible for individuals within the same BMI category to have significantly different amounts and distributions of body fat, thereby yielding differing health risks. For instance, fat mass is associated with an increased risk of cardiovascular disease mortality in individuals with a normal BMI [53,54]. Trunk fat mass is also a strong indicator of unfavorable metabolic characteristics (e.g., insulin resistance) associated with an increased risk of cardiovascular disease [55]. Furthermore, the %FM in pregnancy is positively associated with blood glucose, blood pressure, and insulin resistance, which are strongly associated with adverse pregnancy outcomes and gestational diabetes [6]. Accordingly, fat mass may play an important role in the onset of cardiometabolic disease and diabetes during pregnancy. Our online HIIT intervention provided a convenient way to exercise at home during the COVID-19 lockdown [56,57] and regulated the growth of fat mass during pregnancy more effectively. Thus, it may help pregnant women maintain a healthy lifestyle and reduce the risk of contracting COVID-19 during social gatherings.

### 4.3. Correlation of the Changes in the Parameters Related to the Anaerobic Threshold and Body Composition with the Characteristics of the Exercise Intervention

The exercise intervention characteristics were associated with changes in several parameters related to the AT. In the HIIT group, the number of HIIT sessions was positively correlated with the changes in the VO_2_/AT; the number of self-performed sessions with the changes in the VO_2_/AT and %VO_2max_/AT; and the total number of sessions with the changes in the HR/AT, VO_2_/AT, and %VO_2max_/AT (Figure 6). In contrast, the number of self-performed sessions was negatively related to the changes in the VO_2_/AT and %VO_2max_/AT among the pregnant women who received only the educational intervention and were encouraged to undertake physical activity on their own. Accordingly, the HIIT program under the guidance of a professional exercise specialist had a stronger effect on exercise capacity. The HIIT group was instructed on how to perform high-intensity exercises and educated about the safety and benefits of HIIT for their pregnancy progression and their unborn child’s development. The fear for the child’s safety was assumed to be a substantial barrier to participating in more intense activities. The use of an online, supervised HIIT intervention combined with education not only guaranteed accurate and safe exercise but was also more effective than educational intervention alone [58]. In our study, the EDU group undertook less intense physical activity (RPE of 5 ± 2 on the 0–10 Borg RPE Scale), which probably limited the effectiveness of the intervention on the exercise-capacity parameters. These findings may serve as a foundation for future recommendations regarding high-intensity exercise during pregnancy.

Notably, the %FM significantly decreased following the 8-week HIIT intervention, but the reduction was not related to the number of HIIT sessions or the intensity of each session. Scientists have speculated that the fat-burning effects of HIIT may not be attributed to the direct burning of fat during HIIT but rather to the body’s increased ability to metabolize fat during daily activities and exercise. The body obtains most of its metabolic energy from the decomposition of carbohydrates when the exercise intensity is over 85% of the VO_2max_ [58]. In our study, the intensity of the HIIT intervention was expected to be greater than the AT. The interval portion of the HIIT session was probably based at least to some extent on anaerobic glycolysis for energy metabolism. The better exercise-capacity parameters in the HIIT group could be associated with the improved utilization of fat during routine daily activities, thereby resulting in a lower %FM after 8 weeks of HIIT.

Another interesting issue that we observed in our study was the different rate of dropout from the study between the HIIT and EDU groups (3% vs. 31%). The women from the HIIT group became very involved in the intervention. Only one participant resigned after a few classes due to family duties. We suspect that women who received the supervised exercise program were significantly more motivated and likely more willing to overcome the barriers to attending classes. Our observations were in line with the works by other authors. For example, Anderson et al. [59] found that pregnant women considered HIIT sessions to be more “interesting” and “challenging” and that they provided a “better workout” and made time “go faster” compared to continuous training. Halse et al. [60] noted that a HIIT cycling program enhanced pregnant women’s attitudes and intentions toward exercise. Training enjoyment is of particular importance because it significantly predicts exercise adherence [61], which consequently may determine desired health benefits. The analysis of the motivation of pregnant women to participate in our HIIT intervention is worthy of further research.

In turn, the high dropout rate in the comparative group may have resulted from the feeling that they did not get what they came to the study for. The EDU group also consisted of women who were potentially interested in physical activity and probably felt disappointed by their allocation to the educational intervention. Apart from the premature labor (which was not related to physical activity or other lifestyle factors) in one participant, other reasons for discontinuation of the intervention reported by the EDU group appeared to be possible to overcome with appropriate incentive strategies and counseling. However, the online lectures once a week did not seem sufficient in this regard.

### 4.4. Strengths and Limitations

To our knowledge, this was the first study to assess the effects of a HIIT program on the parameters related to the AT and body composition of women with uncomplicated pregnancies. The strength of our work is that it evaluated the outcomes of 8-week HIIT intervention. Most of the available studies in human populations assessed the acute effects of a single HIIT session. Longer HIIT interventions lasting for several weeks were conducted in animal models [14]. In addition, the online mode of provision of our interventions (both HIIT and educational) may serve as solutions to maintain a sufficient level of physical activity in pregnant women during future lockdowns as during the COVID-19 pandemic. Outside the pandemic period, the online provision of HIIT programs can be a good solution for pregnant women who have limited access to sport facilities, who lack the time to travel to the gyms, or who need to stay home (e.g., due to family reasons, including taking care of older children).

Nevertheless, our work had some limitations that should be considered when evaluating the conclusions. First, we only recruited pregnant women of a single ethnicity to minimize the degree of heterogeneity among the participants. Despite being likely representative of a demographic and socially similar population, the findings may be limited in their application to other races. Second, no information regarding dietary intake was obtained. In pregnant women, protein supplementation may enhance the beneficial effects of HIIT. Another weak point of our work was that we did not compare our intervention to other online exercise programs. Certainly, our educational group seemed to be an interesting comparative group because it represented pregnant women under standard obstetric care. In accordance with the current guidelines [13], all pregnant women should obtain information on a healthy lifestyle, including on physical activity, from their obstetric care providers. However, the comparison of the effectiveness of online prenatal HIIT to an online moderate-intensity continuous program would be very valuable.

Further research is needed to investigate the above-mentioned issues. In future work, it would be also interesting to estimate the impact of our HIIT intervention in those pregnant participants who performed less than 70% of the entire exercise program; e.g., using intention-to-treat (ITT) analyses with linear interpolation. Another valuable question regards how long the better exercise capacity outcomes remain after delivery. Such an analysis could have a practical value in developing recommendations on the implementation of pre-natal and post-natal HIIT programs.

## 5. Conclusions

Our 8-week online HIIT program had a positive impact on the exercise capacity and the body composition in women with uncomplicated pregnancies without producing adverse obstetric and neonatal effects. Despite physiological pregnancy weight gain and pregnancy progression, after the HIIT intervention the parameters related to the AT were better or maintained at the same level. What is more, the %FM decreased in this group. Our findings indicated that online, supervised HIIT combined with education on a healthy lifestyle during pregnancy had a greater impact on health parameters than education alone. It seems likely that similar interventions are necessary for pregnant women with multiple or complicated pregnancies or other races if HIIT programs are to be popularized widely among pregnant women. This online protocol can potentially promote exercise programs during the COVID-19 pandemic and in situations where women have limited time or access to sport facilities.

## Figures and Tables

**Table 1:** The characteristics of HIIT intervention.

The Characteristics of HIIT Intervention					
Week Number	Time of Workout Interval (s)	Time of Rest Interval (s)	Number of Sets (Workout + Rest Intervals)	Time between Sets (s)	Number of Cycles (Exercises)
Week 1	30	60	4	60	4
Week 2	30	60	4	30	4
Week 3	45	45	4	60	4
Week 4	45	45	4	45	4
Week 5	45	45	4	30	4
Week 6	30	30	4	60	4
Week 7	30	30	4	30	4
Week 8	30	15	4	30	4

**Table 2:** Comparison of the baseline parameters related to the anaerobic threshold between the HIIT and EDU groups.

Parameters Related to the Anaerobic Threshold at Baseline	Group (Mean ± SD ^6^)			
	EDU ^2^ (*n* = 21)	HIIT ^3^ (*n* = 28)	*p*	Cohen’s *f*
HR/AT ^1^ (beat⸳min^−1^)	151 ± 10	153 ± 9	0.56	0.09
Time between the HR/AerT ^4^ and HR/AT ^5^ (s)	92.76 ± 24.19	79.48 ± 29.83	0.10	0.24
Time above the HR/AT (s)	140.74 ± 52.05	128.07 ± 47.83	0.38	0.13
VO_2_/AT ^7^ (mL⸳kg^−1^⸳min^−1^)	19.13 ± 2.86	20.38 ± 2.93	0.14	0.22
%VO_2max_/AT ^8^ (%)	79.18 ± 7	78.96 ± 7	0.16	0.20

^1^ AT: anaerobic threshold, ^2^ EDU: educational, ^3^ HIIT: high-intensity interval training, ^4^ HR/AerT: heart rate at the aerobic threshold, ^5^ HR/AT: heart rate at the anaerobic threshold, ^6^ SD: standard deviation, ^7^ VO_2_/AT: maximal oxygen uptake at anaerobic threshold, ^8^ %VO_2max_/AT: percentage of maximal oxygen uptake at the anaerobic threshold. Data were analyzed using one-way ANOVA.

**Table 3:** Baseline body composition in the HIIT and EDU groups.

Body Composition	Group					
	EDU ^2^ (*n* = 21)		HIIT ^3^ (*n* = 28)		*p*	Cohen’s *f*
	Median	Range (Min–Max)	Median	Range (Min–Max)		
BMI ^1^ (kg⸳m^−2^)	25.23	(22.4–27.5)	23.75	(22.4–26.9)	0.11	0.24
Weight (kg)	70.90	(65.1–78.3)	67.10	(61.8–72.0)	0.17	0.20
%FM ^5^	32.9	(30–40)	30.3	(30–30)	0.14	0.22
%FFM ^4^	67	(60–70)	70	(70–80)	0.14	0.22

^1^ BMI: body mass index, ^2^ EDU: educational, ^3^ HIIT: high-intensity interval training, ^4^ %FFM: fat-free mass percentage, ^5^ %FM: fat mass percentage.

## Data Availability

The datasets generated during and/or analyzed during the current study are available from the corresponding author (H.Y.) or the project head (A.S.) upon reasonable request.
